# Costs of treating cardiovascular events in Germany: a systematic literature review

**DOI:** 10.1186/s13561-015-0063-5

**Published:** 2015-09-23

**Authors:** Tamara Schmid

**Affiliations:** Amgen GmbH, Hanauer Str. 1, 80992 Munich, Germany

## Abstract

**Objective:**

This study aims to systematically evaluate available evidence regarding direct medical costs of treating cardiovascular (CV) events in Germany after 2003 on an individual patient basis and from a payer perspective. The CV events of interest were myocardial infarction (MI), unstable angina, heart failure (HF), stroke, and peripheral artery disease (PAD).

**Method:**

A systematic literature search was performed in the following databases according to Preferred Reporting Items for Systematic Reviews and Meta-Analysis (PRISMA) guidelines - Medline, Embase, Centre for Reviews and Dissemination, TIBORDER, and German dissertation database from January 2003 to October 2013. Both observational studies and randomized clinical trials were considered for the review. All values stated in € are inflation adjusted to 2014 € unless stated otherwise.

**Result:**

This review included 13 articles. For newly occurred MI patients, the average hospitalization costs during the acute phase were reported to be between € 6790 and € 8918 per admission. In the first year after a MI event, direct medical costs were € 13,838–14,792 per patient. Direct medical costs of chronic HF patients were found to be between € 3417 and 5576 per patient per year. Treatment costs increase with disease progression. The average treatment costs for hospitalized PAD in the acute phase were reported to be € 4963 per admission, € 2535 per patient during month 1–6 after the initial hospitalization, € 1601 in month 7–12, and € 1390 in month 13–18. For stroke of all types, total direct medical costs in the 1st year after an event were reported to be € 13,273 per patient. Total direct medical costs during the 1st year after an ischemic stroke event were € 17,399–21,954 per patient, € 6260 in month 13–18, and € 6496 per year in the subsequent 4 years.

**Conclusion:**

MI, unstable angina, HF, stroke and PAD have a high financial impact on the German health care system. Treatment costs of these diseases are mostly incurred during the acute phase of events and tend to decrease over time. Hospitalization and rehabilitation costs were two major cost drivers. Medication costs was one of the smallest cost component reported.

## Review

Cardiovascular (CV) events are mainly disorders of heart and blood vessels, including coronary heart disease (CHD), cerebrovascular disease (mainly stroke), and peripheral artery disease (PAD). In 2012, CHD is the leading cause of death worldwide, with an estimated 7.4 million people died from it followed by 6.7 million died from stroke. In Germany in 2012, CHD was number one cause with a proportion of 40,2 % on overall death [1, 2].

Acute CHD, stroke, and PAD often require intensive treatments, such as coronary artery bypass grafting (CABG), percutaneous coronary intervention (PCI) and/or fibrinolysis. Often patients experiencing these disorders also need long-term treatment and care, as well as management after the acute stage. All these treatments and procedures lead to a tremendous economic burden to the patients, third party payers, and the society as a whole.

In Germany, CV event is highly incident and is one of the most expensive diseases. DEGS1 studies (Results of the German Health Interview and Examination Survey for Adults) from 2013 discovered a lifetime prevalence of overall CHD in Germany of 9.3 % among women and men aged 40 to 79 years [3]. According to the 2008 Federal Health Report published by the Federal Statistical Office of Germany, the costs caused by CV events amounted to € 35.5,000,000,000, which was 1/6 of the total expenditure of healthcare provision in Germany in that year [4]. Moreover, CHD, angina pectoris, stroke, and MI are among the 20 leading causes for hospitalization [5]. 40 % of people in Germany die due to cardiovascular diseases. Among all CV events, cerebrovascular diseases such as stroke accounted for € 7.8,000,000,000, ischemic heart disease such as cardiac infarction for € 7,000,000,000, and heart failure for € 2.7,000,000,000 in 2008 [6].

However, there is a gap in publications with respect to the individual treatment costs of CV events, especially after the implementation of German diagnostic-related groups (G-DRG) in hospitals in 2003 [7], which gave healthcare providers incentives to shorten the length of stay and be more cost-conscious [8].

This systematic literature review aims to provide an overview of the costs of treating individual CV event patients after 2003 from a third-party payer perspective. The CV events of interest were myocardial infarction (MI), unstable angina, heart failure (HF), stroke, and PAD.

## Methods

### Identification of studies

A systematic literature search was conducted to identify all available publications about individual-level direct medical costs of the CV events of interest in Germany after the G-DRG reform. We adopted the perspective of healthcare providers and German social third party payers, including statutory health insurance (SHI) and statutory nursing insurance (SNI) for cost studies. The scope of the costs was restricted to direct medical costs per affected patient, including hospitalization costs (based on the G-DRG system), outpatient costs (based on the points assigned to the particular treatment published in the German tariff list), medication costs (based on the reimbursement price of German SHI, rather than the price set by manufacturers), and rehabilitation and ambulance/paramedic costs (based on the costs reimbursed by the German SHI or SNI).

The review was conducted according to the Preferred Reporting Items for Systematic Reviews and Meta-Analyses (PRISMA) guidelines [9].

Publications between January 1^st^ 2003 and October 1^st^ 2013 in English or German were searched in the following databases: Medline, Embase, Centre for Reviews and Dissemination (CRD) database, TIBORDER database, and German dissertation database.

Published papers, conference abstracts, and dissertations were all included in this review. Search terms are presented in Table 1.

**Table 1:** Search terms for the systematic literature review^a^

Domain	Search terms
Subject	“cost” OR “costs” OR “expenditure” OR “economic” OR “burden” OR “resource”
Setting	“German” OR “Germany” [full text]
Cardiovascular events	“myocardial infarction” OR “myocardial infarction” [MeSH]
	“unstable angina” OR “angina, unstable” [MeSH]
	“heart failure” OR “heart failure” [MeSH]
	“peripheral vascular disease” OR “peripheral vascular event” OR “peripheral artery disease” OR “peripheral vascular diseases” [MeSH]
	“stroke” OR “stroke” [MeSH]

^a^The terms are searched in title/abstract if there is no additional explanation

Observational, epidemiological studies and randomized clinical trials (RCTs) with individual cost information were included. For RCT publications, the RCT was included in the review if there was original information about costs. References of the RCT studies were reviewed by hand for sources of original cost data.

Exclusion criteria were: setting was not Germany; not SHI perspective or healthcare provider perspective adopted; not including the CV events of interest; costs data before 2003; costs on individual level not available or not calculable; costs reported originated from other studies (in this case the cited study was hand-searched and reviewed); and costs evaluation using health economic models or truncated approaches (no direct data).

One researcher conducted the search. If there was doubt regarding the inclusion/exclusion of an article, another independent researcher was consulted.

All values stated in € are inflation-adjusted to 2014 € unless stated otherwise.

### Data extraction and analysis

Relevant data was extracted in a Microsoft Excel file. The extracted parameters included study identification, study characteristics (i.e. objective, setting, perspective), type of CV events, intervention (i.e. % of each cardiovascular treatments, such as drug treatment, PCI, CABG, etc.), participants characteristics (i.e. gender, age, comorbidities), direct medical costs (including cost in acute phase, costs within the first year after the event and follow-up costs from the second year onwards), conclusion and limitations of the study.

## Results

The search resulted in 400 hits. The reviewer removed 50 duplicated papers, leaving 350 papers for title and abstract-based selection. There were 64 articles/abstracts (including 4 additional papers based on hand search from citations of relevant papers) included for full-text screening, among which 51 articles/abstracts were excluded based on the pre-defined exclusion criteria: 9 studies were excluded because the reported CV event (e.g. atherothrombosis) was not of interest for this study; 3 because German costs were not separately reported; 24 because they failed to report individual costs, instead they reported incremental costs of interventions compared to base treatments; 8 due to lack of individual cost data; 4 studies reported costs based on data before 2003; 3 studies reported costs using a model or using truncated approach based on indirect data. Ultimately, there were 13 studies included in this review, with several studies addressing multiple CV events.

**Fig. 1:**
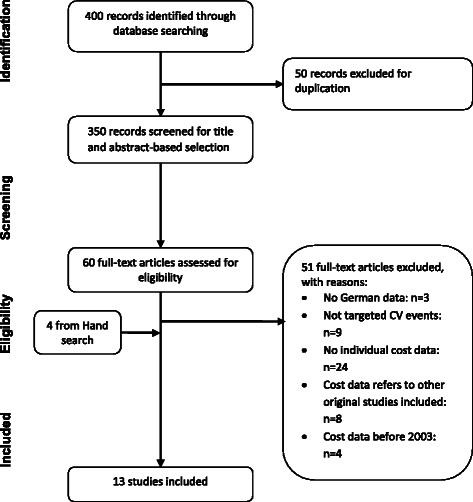
Flow chart of included studies

The selection procedure was recorded following the PRISMA flowchart (Fig. 1).

### Myocardial infarction

Seven papers and one abstract reported direct medical cost of individual MI patients in Germany [10–17].

In the literature, methods used to investigate costs of MI include a claims data analysis, a hospital database analysis, medical chart review, and calculations of costs based on healthcare resource utilization from expert panels (or literature review and expert interview) and SHI price. Although there was no clear definition in the included articles regarding acute and long-term treatment of MI, treatment costs can be roughly categorized into costs during acute phase (or hospitalization costs), costs within 1 year after event, and long-term costs after event (normally from the 2^nd^ year onwards).

For newly occurred acute myocardial infarction (AMI) patients, the average hospitalization costs during acute phase ranged from € 3622 to 8918/patient [11, 13, 15–17].

During the first year after an AMI event, direct medical costs of treating AMI patients were reported to be from € 13,838 to 14,792/patient [10, 17]. Total costs spent on male patients were about 21 % higher than those for females (*p* ≤0.0001) [13]. Treatment costs seemed to decrease over time during the 1^st^ year after an event. As estimated by Brüggenjürgen and Reinhold, 50–64 % of total costs in the first year were spent on treatment within the acute phase, and 80–85 % of total cost estimates were reached within 6 months after the event [13, 17]. According to the study conducted by Brüggenjürgen et al. hospitalization costs covered the largest share (69 %) of total first-year costs, followed by rehabilitation costs (14 %) and a relatively small proportion on medication costs (8 %) [17].

Treatment costs were heavily influenced by treatment strategies and the medical devices used. For example, treatment costs of stent implementation depended on the type of stent used: the use of bare-metal stent (BMS) led to less resource use than drug-eluting stent (DES) (€ 13,629 vs. 14,792, *p* <0.05), with more than 50 % of costs occurring within 30 days after the implementation [10]. Among patients who underwent PCI, in-hospital treatment costs of contemporary antithrombotic strategies (between € 3545 and 4755, roughly 1/3 of the total treatment costs in the first year) were highly related to the anticoagulant drug(s) used [12]. Inpatient cardiac rehabilitation for MI in the first year was also a significant cost component of the total treatment costs, estimated to be € 2304/patient (around 14 % of the total 1^st^ year costs) [14].

Direct treatment costs decreased to € 1163/patient during month 13–18 after an AMI event [17].

### Unstable angina

One study based on claims data was identified that examined the direct medical costs of treating unstable angina [18].

The published average costs for the inpatient treatment per unstable angina patient (acute phase) were € 4442, with inpatient cardiac rehabilitation being cheaper than inpatient treatment only [18]. For hospital-admitted patients, the leading cost drivers were hospitalization costs (€ 2414), followed by angiography costs (€ 829), diagnostic costs (€ 683), and drug costs (€ 391, equal to 9 % of the total costs) [18].

### Heart failure

Two studies reported costs of treating HF from a SHI perspective in Germany [19, 20]. Both were database analyses.

Direct medical costs of CHF were calculated to be between € 3417 and 5576/patient per year for patients of all New York Heart Association (NYHA) levels [19, 20]. Biermann et al. found that the largest cost component was related to hospitalizations (€ 2525, 74 %); while costs of rehabilitation (€ 319, 9 %), medication (€ 315, 9 %) and outpatient contacts (€ 258, 8 %) were considerably less important cost components [19]. Compared with a cost of € 2683 per patient per year for NYHA level I patient, there was a cost increase in NYHA II, III and IV of 14, 48, and 71 %, respectively. About 76 % of the cost increase resulted from augmented hospital (inpatient) resource use [19].

### Stroke

The review resulted in 5 papers regarding the direct medical costs of treating stroke in Germany [16, 17, 21–23]. Different methods were applied in these studies, including claims data analyses, calculations based on resource utilization and SHI price, and retrospective cost-of-illness study.

For stroke of all types, total direct medical costs in the 1st year after an event were reported to be € 13,273 perpatient [22]. Male patients incurred approximately 10 % more costs than females. About half of the costs were incurred in the first 4 weeks after the event, 80 % of the costs were reached in the first 6 months [16, 22]. Hospitalization costs accounted for the largest share (75 %) of the total costs of treating stroke [22].

For ischemic stroke, total direct medical costs in the 1^st^ year after the event were € 17,399–21,954 per patient [17, 21, 23]. 29 % of the total costs in the 1^st^ year incurred in the acute phase, 66 % were reached within 6 months after the event [17]. 1^st^ year treatment costs for ischemic stroke patients varied due to the treatment settings where they received rehabilitation treatments: hospitalized patients had around € 3352 more costs per patient per year compared to those treated in rehabilitation centers [21]. For patients admitted to a hospital for post-stroke rehabilitation, the leading components of 1^st^ year costs was hospitalization costs (€ 13,953), followed by medicines (€ 1915) and outpatient care (€ 1066). For patients who were admitted to a rehabilitation facility for post-stroke rehabilitation, the leading components were hospitalization costs (€ 8844), inpatient rehabilitation care (€ 4788), medicines (€ 1691), and outpatient care (€ 1317). For both groups, hospitalization (including rehabilitation) is the main cost driver for rehabilitation treatment of ischemic stroke patients [21]. Rehabilitation accounted for the largest share (37 %) of costs in the first year after an ischemic stroke event [23].

In month 13–18, costs of treating ischemic stroke were € 6260 per patient [17]. During year 2 to 5 after the event, direct medical costs decreased to € 6496 per patient per year [23].

No cost information regarding hemorrhagic stroke and transient ischemic attack was identified.

### Peripheral artery disease

Only one paper was identified reporting direct medical costs of treating PAD [17].

Using a Delphi panel to obtain healthcare resource utilization and calculating costs based on reimbursed price, Brüggenjürgen et al. reported that the average treatment costs for hospitalized PAD in the acute phase (hospitalization) were € 4963 per admission, € 2535 per patient during month 1–6 after the initial hospitalization, € 1601 in month 7–12, and € 1390 in month 13–18 [17], meaning that 54.5 % of the 1^st^ year costs are consumed in the acute phase and 82.4 % within the first 6 months.

The key results of all included studies are presented in Table 2.

**Table 2:** Direct medical costs of several cardiovascular diseases in Germany (2003–2013) from a healthcare provider or SHI perspective

Study	Disease	Study design	Sample size	Gender (% male)	Age (mean ± SD years)	Unit of costs	Costs in the acute phase (actual costs; % of costs in the 1st year)	2014 € Inflation adjusted costs in acute phase^a^	Costs/patient in the first 6 months (actual costs; % of the costs in the 1st year)^b^	2014 € Inflation adjusted costs/patient in the first 6 months^a^	Costs/patient in the 1st year after event	2014 € Inflation adjusted costs/patient in the 1st year after event^a^	Costs/patient in subsequent years	2014 € Inflation adjusted Costs/patient in subsequent years^a^	Other costs	2014 € Inflation adjusted other costs^a^	Note
Myocardial infarction and unstable angina																	
Stargardt 2013 [11]	MI	Retrospective claim database analysis	12,284	71.60 %	64.1	2004–2006 €	n.a.	n.a.	n.a.	n.a.	n.a.	n.a.	n.a.	n.a.	Hospitalization costs: 6936	Hospitalization costs: 7927	Used provider reimbursement for costs
Reinöhl 2012 [12]	AMI	Real-world scenario administrative routine data analysis (cross sectional study)	1409	n.a.	n.a.	Not stated in publication, assume year of publication 2012.	n.a.	n.a.	n.a.	n.a.	3461.82–4643.15 depending on different anticoagulant strategy used for PCI treatment	3545–4755 depending on different anticoagulant strategy used for PCI treatment	n.a.	n.a.	In-hospital costs:	In-hospital costs:	Only patients underwent PCI at high volume centers
															^a^Using UFH monotherapy: 3807.2 ± 2235.98;	^a^Using UFH monotherapy: 3899 ± 2290;	
															^a^UFH + glycoprotein IIb/IIIa receptor inhibitor: 4643.15 ± 4662.48;	^a^UFH + glycoprotein IIb/IIIa receptor inhibitor: 4755 ± 4775;	
															^a^Bivalirudin: 3461.82 ± 1301.96	^a^Bivalirudin: 3545 ± 1333	
Bäumler 2012 [10]	MI	Retrospective claims data analysis	DES: 719; BMS: 719	DES: 86.20 %; BMS: 86.07 %	DES: 60.7 (11.2); BMS: 62.2 (11.0)	2005	30 day (acute) costs were 7761 for DES group and 6704 for BMS group	30 day (acute) costs were 9030 for DES group and 7800 for BMS group	n.a.	n.a.	DES: 12,713 (SD 10,753); BMS: 11,714 (SD 9967)	DES: 14,792 (SD 12,511); BMS: 13,629 (SD 11,597)	n.a.	n.a.	n.a.	n.a.	Only patients with PCI; First MI only, excluded re-infarct
Reinhold 2011 [13]	MI	Retrospective claim data analysis	15,185	57.43 %	71.1 ± 12.6	2004/2005 €	5836; 50 %	6790	9897; 80 %	11,515	12,372	14,395	n.a.	n.a.	n.a.	n.a.	Largest SHI with representative sample; excluded recurrent events
Tiemann 2008 [15]	AMI	Retrospective study using hospital administration data	n.a.	100 %	50–60	2005	3113 from SHI perspective; 2866 from hospital perspective	3622 from SHI persp.; 3335 from hospital persp.	n.a.	n.a.	n.a.	n.a.	n.a.	n.a.	n.a.	n.a.	Selective patient group (i.e. no revascularization, no teaching hospitals)
Fuchs 2008 [16]	MI	Calculate costs based on expert interview resource utilization data/or published data and German SHI price	n.a.	n.a.	Assume 70 % over 60 years old	2005	4560	5306	n.a.	n.a.	n.a.	n.a.	n.a.	n.a.	n.a.	n.a.	No information on resource use estimates
Brüggenjürgen 2006 [18]	Hospitalized UA	Prospective cross-sectional study in 19 hospitals of different health care levels	407	67.1 %	65.9 (11.6)	2000–2002 €	3644 (SD 2195, 95 % CI: 3430–3858)	4442 (SD 2676, 95 % CI: 4181–4703)	n.a.	n.a.	n.a.	n.a.	n.a.	n.a.	n.a.	n.a.	n.a.
Brüggenjürgen 2005 [17]	MI	Calculate costs based on expert panel resource utilization data and German SHI price	n.a.	n.a.	n.a.	2004	7522; 64 %	8918	85 %	n.a.	11,672	13,838	981 during month 13–18 after the event	1163 during month 13–18 after the event	n.a.	n.a.	Expert panel stated 57 % PTCA, 7 % CABG, 7 % pacemaker, 69 % rehab
Chronic heart failure																	
Biermann 2012 [19]	CHF	Retrospective panel study using claims data	2,71	74.8 %	62.9 ± 13.6	2009	n.a.	n.a.	n.a.	n.a.	3150	3417	n.a.	n.a.	Inpatient care: 74 %	n.a.	Included NYHA I-IV
															Medication: 9 %;		
															Rehabilitation: 9 %		
															outpatient contact: 8 %		
Peters-Klimm 2012 [20]	CHF	Retrospective medical chart analysis	159	73 %	68.5 ± 10.2	2004–2005 €	n.a.	n.a.	n.a.	n.a.	4792 ± 8249	5576 ± 9598	n.a.	n.a.	Total hospitalization: 3545 (8065), including HF 466 (1525), other CV-related hospitalization 2596 (7469), and other causes of hospitalization 483 (1894);	Total hospitalization: 4125 (9384), including HF 542 (1774), other CV-related hospitalization 3020 (8690), and other causes of hospitalization 562 (562);	53 % NYHA II, 45 % NYHA III; Based on a RCT regarding an innovative medical education on primary care-based patients
															Medication: 854 (835)	Medication: 994 (972)	
Peripheral artery disease																	
Brüggenjürgen 2005 [17]	PAD	Calculate costs based on expert panel resource utilization data and German SHI price	n.a.	n.a.	n.a.	2004	4186; 55 %	4963	2138; 28 %	2535	7674	9098	1172 during month 13–18 after the event	1390 during month 13–18 after the event	2138 for month 1–6; 1350 for month 7-12	2535 for month 1–6; 1601 for month 7–12	Only hospitalized patients included
Stroke																	
Abbas 2013 [21]	Ischemic Stroke	Claims data analysis	Hospital based: 1272;	28 % hospital-based, 33 % for facility	80–81 years	2007	n.a.	n.a.	n.a.	n.a.	Hospital based: 15,573; rehabilitation facility based: 15,726	Hospital based: 17,399 rehabilitation facility based: 17,570	n.a.	n.a.	n.a.	n.a.	Rehab patients only
			Rehabilitation facility based: 2200														
Lindig 2010 [22]	Stroke	Retrospective claim data analysis	18,106	43.6 %	73.7 ± 12.6 years	2004/2005 €	Around 50 %	n.a.	Around 80 %	n.a.	11,408	13,273	n.a.	n.a.	n.a.	n.a.	All hospitalized patients; includes hemorrhagic & ischemic strokes
Fuchs 2008 [16]	Ischemic stroke	Calculate costs based on expert interview resource utilization data/or published data and German SHI price	n.a.	n.a.	Assume 70 % over 60 years old	2005	4780	5562	n.a.	n.a.	n.a.	n.a.	n.a.	n.a.	n.a.	n.a.	n.a.
Kolominsky-Rabas 2006 [23]	Ischemic stroke	Retrospective cost of illness study based on a population-based longitudinal registry database and German SHI prices.	821	45 %	52 % patients were in the age group >75 years, women (76.3), men (70.6)	2004	n.a.	n.a.	n.a.	n.a.	18,517	21,954	Annual costs for subsequent 4 years were 5479/patient.	Annual costs for subsequent 4 years were 6496/patient.	n.a.	n.a.	First-year survivors after first-ever stroke
Brüggenjürgen 2005 [17]	Ischemic stroke	Calculate costs based on expert panel resource utilization data and German SHI price	n.a.	n.a.	n.a.	2004	5134; 29 %	6087	6727; 66 %	7976	17,864	21,180	5280 during month 13–18 after the event	6260 during month 13–18 after the event	6003 for months 7–12	7117 for months 7–12	Experts estimated 53 % rehab facility, 45 % inpatient rehab

Note: “Costs” in this table is limited to direct medical costs from a SHI perspective in Germany. n.a. means not available. n.r. means not relevant

^a^Inflation adjusted costs according to CPI: OECE statistics extracts, http://stats.oecd.org/Index.aspx?DataSetCode=MEI_CPI_WEIGHTS#, accessed on AUG 10 2015

^b^The costs incurred in the first 6 months include those incurred in the acute phase

## Conclusion

**Fig. 2:**
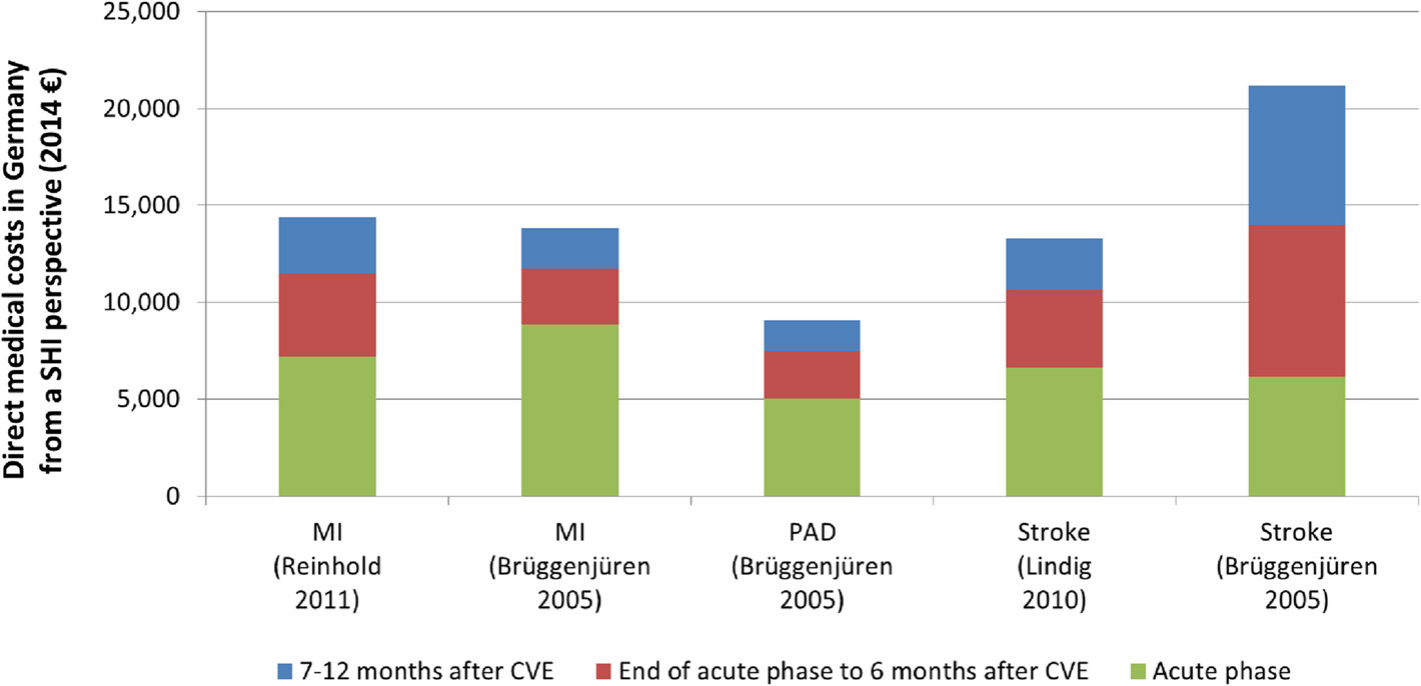
Costs in treating MI, PAD, and stroke in the first year after CVE. Abbreviations: CVE, Cardiovascular Events, MI, Myocardial Infarction, PAD, Peripheral Artery Disease, SHI, Statutory Health Insurance

To summarize, MI, unstable angina, HF, stroke and PAD are leading to high direct medical costs in Germany. Treatment costs of MI, PAD and stroke mostly concentrate during the acute phase of the events and tend to decrease over time. Figure 2 shows the results of the studies that reported treatment costs in different time frames. Hospitalization costs and rehabilitation costs are two major cost drivers for all CV events of interest. This suggests that it might be cost-saving to increase investment into the prevention of these CV events in order to avoid costly future hospitalization.

## Discussion

This study is, to our knowledge, the first systematic literature review of direct medical costs of treating CV events on individual patient’s level in Germany.

As what was found in this review, treatment costs of CV events are high in developed countries. However, the results are not comparable across studies from different countries because of potential various treatment patterns, reimbursement systems, different study years, etc. For example, it was reported that the treatment costs of AMI during 6 months post event were $ 2764–4953 (equal to 2014 € 2436–4365 [1]) in the US in 2005 [24]. In Sweden, the average costs of HF-related hospitalizations was SEK 72,613 (2014 € 8156) per patient per year; in contrast, annual prescription costs were low: on average 3 % of total cost (SEK 3503, 2014 € 393 per patient) [25]. Similar to that in Germany, in the US stroke seems to be the most expensive CV event among those included in this study. Based on 2.7,000 000 hospital admissions with a diagnosis of stroke in the US, the average total charge per admission were $ 46,518 (equal to 2014 € 35,202^1^) in 2009 [26].

In Germany, although treatment costs of CV events are overall high, there was a substantial variance (around € 1000/patient per year) in the costs reported, especially in stroke treatment (up to € 3000/patient per year). Reported costs varied across studies due to the following reasons: 1) different study design, 2) different patient group, and 3) different treatment pattern.

Among the 13 studies identified in this review, research methods adopted included claims data analyses, which included relatively large sample sizes from multiple healthcare providers [10, 11, 13, 21, 22]; retrospective observational studies based on a few cardiovascular centers [12]; retrospective patient registry analyses, which composed of various RCTs and cohorts [19, 23]; from expert interviews or medical chart review [16, 17]; and RCTs [14, 20].

As an example, Brüggenjürgen et al. [17] reported € 3780 and 4496 per patient more cost estimation for a stroke patient during the end of acute phrase to 6 months after event and 7–12 months after event, respectively, compared to Lindig et al. [22] (see Fig. 2). As the Lindig study is an observational one based on claims data, it represented the real costs in the German practice. The Brüggenjürgen study was based on experts’ opinion regarding resource utilization and calculation using published price; it might represent the “recommended” treatment pattern and costs accordingly.

In general, results of RCTs are least representative for the treatment costs of general CV patients in Germany as they normally have strict recruitment procedures based on patients’ baseline characteristics, health problems and medical history [27]. Results of the observational studies, including only a few high-volume cardiovascular centers, also need to be interpreted with caution as these hospitals are likely to treat more severe patients (referred to by secondary hospitals) leading to higher resource use and costs per case. Moreover, as all of these studies [12, 14, 20] evaluated the treatment costs of specific treatment strategies, e.g. use of anticoagulants, out- and inpatient rehabilitation, and a medical education program. With these differences across studies, it is difficult to ascertain representative data for direct medical costs in Germany.

Due to different study objectives, researchers had variant inclusion and exclusion criteria (e.g. age, new disease vs. recurrent disease, disease of different levels, etc.) for cohorts. For example, Tiemann investigated the costs of primary treatments of AMI patients to compare variations in hospitalization costs over 9 European countries, including Germany [15]. In order to compare costs across countries; selection of patients was strict in this study. Only male patients between 50 and 60 years old without relevant co-morbidities (e.g. diabetes, hypertension, congestive heart failure, HIV infection) were eligible. Furthermore, patients were only included in the study if they came to an emergency department within 2 h of symptom(s) onset. Furthermore, in order to avoid the influence of very expensive procedures on the overall results, patients who needed bypass surgery and PCI with complications were also excluded from the analysis. Teaching hospitals have not been included because they might have greater resource intensity and higher costs per case. Due to the extensive restrictions in patient selection, the results of this study can hardly be generalized to all AMI patients in Germany.

Restrictions of patient groups were also implemented in several other studies. For example, Reinhold et al. and Kolominsky-Rabas et al. only included patients who experienced their first-ever event (AMI and stroke, respectively) and excluded recurrent events in their studies respectively [13, 23]. How this restriction influences direct medical costs per case is not clear. In examining the costs of treating MI during acute phase, Fuchs et al. [16] made an assumption of a relatively old patient group for conducting the interviews, which makes their results less generalizable to a wider (younger) patient population.

Different mixes of CHF patients were included in the analyses with respect to NYHA level. The studies of Biermann et al. [19] included patients of all four NYHA categories, while the study of Peters-Klimms [20] included 98 % of NYHA II or III patients. As it was found by Biermann et al. [19] direct medical costs per case increased with severity of HF, the results based on patient groups of a certain NYHA level(s) might not be generalized to patient of other NYHA levels.

Different treatment patterns might influence the cost estimates reported in the studies. To compare the costs and effectiveness of different stents (DES and BMS), Bäumler et al. [10] only included patients who underwent PCI treatment due to their first incidence of AMI. As a result, the findings of this study do not represent costs of the general AMI patient population in Germany.

In order to compare resource utilization of ischemic stroke patients who underwent rehabilitation in a hospital-based unit or geriatric rehabilitation unit, Abbas et al. [21] only included patients who underwent rehabilitation treatment, whereas Lindig et al. [22] included patients who were hospitalized due to stroke. Therefore, costs of outpatient care were not included in these two studies.

Treatment costs are likely to be different across treatment settings (i.e. hospital, rehabilitation center, outpatient setting). For some diseases, such as AMI which is an acute and life-threatening event and most patients are admitted to a hospital, the results of studies including only hospitalized patients are likely to represent all patient groups. For example, the restriction of the study conducted by Stargardt et al. as inpatient only should not have significant influence on the representativeness of the result of this study [11]. However, for some diseases which can be treated in all settings, e.g. chronic heart failure, restrictions of treatment setting might have influence on treatment pattern and consequently treatment costs. For example, Biermann et al. included treatment in both primary and hospital settings [19]; while Peters-Klimm et al. only included patients treated in primary care setting [20]. The results of these two studies are therefore not comparable.

Despite the general awareness of the economic impact of CV events in Germany, the number of observational studies investigating the per-patient direct medical costs is relatively small. It should be noted that no study was identified for the costs of treating acute HF patients, haemorrhagic stroke, and transient ischemic attack from this literature review. There is a need for more research in this area. This literature review is limited to published studies through October 2013. Future literature reviews should be able to include more recent information on CV costs in Germany.

## Footnotes

1 The midpoint exchange rate of Euro/USD was 0.7576 in 2005 and 0.6977 in 2009. Source: www.oanda.com.
