# Erratum to: ‘Costs of treating cardiovascular events in Germany: a systematic literature review’

**DOI:** 10.1186/s13561-016-0080-z

**Published:** 2016-01-07

**Authors:** Tamara Schmid, Weiwei Xu, Shravanthi R. Gandra, Galin V. Michailov

**Affiliations:** 1 Amgen GmbH, Munich, Germany; 2 Pharmerit International, Rotterdam, The Netherlands; 3 Amgen Inc., Thousand Oaks, CA USA

Unfortunately, the original version of this article [1] contained an error. The full author list and affiliations was not included. The correct author list and affiliations can be found below.

Tamara Schmid^a^, Weiwei Xu^b^, Shravanthi R Gandra^c^, Galin V Michailov^a^

a Amgen GmbH, Munich, Germany

b Pharmerit International, Rotterdam, The Netherlands

c Amgen Inc., Thousand Oaks, CA, USA
